# Purification, Structural Characteristics, Bioactive Properties, and Applications of *Naematelia aurantialba* Polysaccharides: A Comprehensive Review

**DOI:** 10.3390/molecules30204073

**Published:** 2025-10-13

**Authors:** Ri-Na Wu, Yun-Yang Zhu, Run-Hui Ma, Zhi-Jing Ni, Xiao-Juan Deng, Kiran Thakur, Zhao-Jun Wei

**Affiliations:** 1 School of Biological Science and Engineering, Specialty Food Nutrition and Health Innovation Team of Ningxia Hui Autonomous Region, North Minzu University, Yinchuan 750021, China; 2 School of Food Science and Engineering, Ningxia University, Yinchuan 750021, China

**Keywords:** *Naematelia aurantialba* polysaccharides, extraction and purification, structural features, bioactivities, potential applications

## Abstract

Jin’er (*Naematelia aurantialba*), commonly known as golden ear, is a traditional edible fungus that has long been recognized for its medicinal and culinary properties in China. Recently, it has been registered as a new cosmetic ingredient, drawing significant attention across various fields, including medicine, food, and cosmetics, due to its array of nutritional and medicinal benefits. *N. aurantialba* is rich in bioactive compounds, such as polysaccharides, dietary fiber, polyphenols, and active peptides. Among these, *N. aurantialba* polysaccharides (NAPs) are the primary active components, exhibiting a range of biological properties, including antioxidant, hypoglycemic, immunomodulatory, intestinal flora modulatory, anti-tumor, and anti-inflammatory effects. This comprehensive review summarizes the latest advancements in the extraction, purification, structural characteristics, functional activity, and related functional mechanisms of NAPs, as well as their industrial applications. Additionally, it discusses the current limitations in NAPs research and explores its potential future research directions. This review aims to provide up-to-date information and valuable references for researchers and industry professionals interested in the potential application of NAPs in the fields of food, medicine, healthcare, and cosmetics.

## 1. Introduction

**Figure 1:**
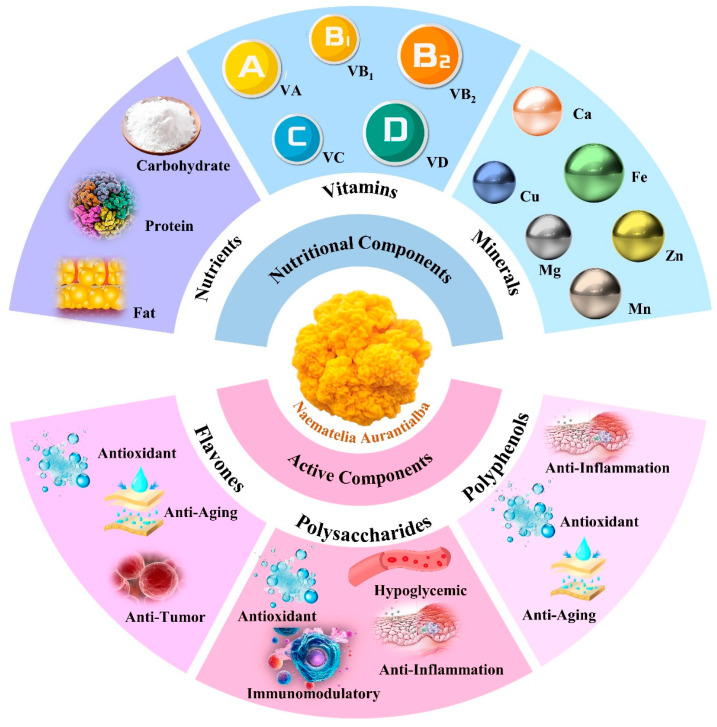
Overview of the nutritional and active components of *Naematelia aurantialba* Polysaccharides (NAPs), highlighting their potential health benefits.

Jin’er (*Naematelia aurantialba* (Bandoni & M. Zang) Millanes & Wedin), abbreviated as *N. aurantialba*, is a rare and precious edible and medicinal fungus native to China. It is commonly known as golden ear, brain ear, or golden wood ear. This fungus belongs to the order *Tremellales*, family *Naemateliaceae*, and genus *Naematelia* Fries [1,2,3]. Previously initially named *Tremella aurantialba*, following advancements in scientific research and species classification, it has been reclassified into the genus *Naematelia*, leading to its current designation as *N*. *aurantialba* [1,4,5]. The wild strain of *N. aurantialba* is predominantly found in the alpine oak forest belt, where it exhibits parasitic or partially symbiotic relationships with species such as Gramma mucronulatum and Gramma flatus. It is distributed across regions including Xizang, Yunnan, Sichuan, and Gansu provinces in China. The formation of the fruiting body of *N. aurantialba* is influenced by the ratio of *N*. *aurantialba* to *Stereum hirsutum*, complicating its artificial cultivation [6,7]. However, with the advancement of artificial breeding techniques, established technologies for the artificial cultivation of its fruiting bodies have been developed in regions such as Ningxia, Yunnan, and Zhejiang in China [1,8]. Various studies have indicated that *N. aurantialba* is a quality source of essential nutrients, such as proteins, amino acids, polysaccharides, fats, minerals, and vitamins. It is also abundant in functional nutrients such as polysaccharides, polyphenols, and flavonoids, which are commonly found in fungi [9,10,11,12]. The nutritional and bioactive components of *N. aurantialba* are shown in Figure 1. According to the text “Chinese Medicinal Fungi”, *N. aurantialba* is warm with a hint of cold in nature and sweet in taste. It is used to treat conditions such as lung heat, cough, asthma, hypertension, and other ailments [4,11]. Given its unique flavor, rich nutritional value, and excellent health functions, *N. aurantialba* is regarded as an important raw material in the food industry. It is frequently used as an active component in functional foods, including vegetable products, fruit processing, and dietary supplements [5,9,11,13,14]. In February 2025, *N. aurantialba* was officially registered as a new cosmetic ingredient in China, with many of its chemical components approved for use as cosmetic additives.

Polysaccharides derived from edible fungi have garnered significant scientific interest in recent decades due to their remarkable biological activities [15,16,17,18]. Systematic research on these polysaccharides offers a valuable opportunity to identify and develop innovative therapeutic agents that enhance human health [19,20,21]. Notably, *N. aurantialba* contains exceptionally high levels of polysaccharides, far exceeding those found in *Tremella fuciformis* and even higher than those in *Dendrobium officinale*. Given its dual status as both an edible and medicinal fungus, *N. aurantialba* polysaccharides (NAPs) have become a focal point of extensive scientific research [4,22]. Recent pharmacological studies have demonstrated that NAPs exhibit a wide range of biological activities, encompassing antioxidative, hypoglycemic, anti-tumor, antihypertensive, anti-inflammatory, and immunoregulatory effects. [1,13,14]. NAPs represent a promising area of fundamental research with promising biomedical applications in food science, natural products, pharmaceuticals, and pharmacology [10,23]. At present, many scholars are engaged in investigating the extraction, separation, and purification methods of NAPs and exploring the intrinsic connection between the chemical structure and physiological functions of NAPs [4,23,24].

In recent years, improvements in living standards and medical care have led to a growing emphasis on obtaining nutrients and health components from diet [25,26,27]. *N. aurantialba* is abundant in natural nutrients and bioactive components, particularly its polysaccharides, which are beneficial to human health and comply with the standards for health foods and nutritional supplements [5,7,11]. However, current research on the purification technology, structural characteristics, biological activities, mechanisms of action, and potential applications of NAPs remains insufficient [4,23,28]. Therefore, this review aims to summarize the research progress of NAPs over the years, covering aspects such as their extraction and purification techniques, structural characteristics, biological properties, and mechanism of action. It also addresses the current research status and gaps in the field of edible fungus polysaccharides, promoting their development and utilization in medicine and functional foods.

## 2. Extraction and Purification of NAPs

**Figure 2:**
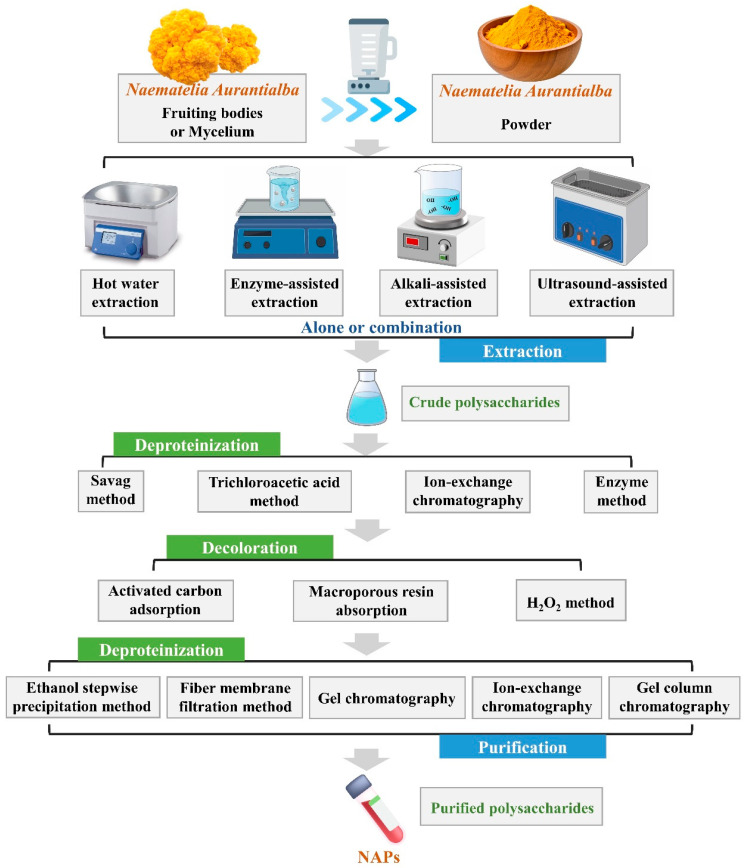
Extraction and purification workflow of NAPs, detailing the initial preparation of the powder, extraction via different methods, followed by deproteinization using various techniques, decolorization, and secondary deproteinization.

Polysaccharides are critical components of *N. aurantialba*, and their extraction and purification form the foundation for subsequent activity research. Prior to extraction, plants typically undergo pretreatment processes such as washing, drying, grinding, sieving, and crushing. The above steps are essential for removing colored contaminants, monosaccharides, oligosaccharides, and other small molecular substances [29,30]. Studies indicated that the extraction method can significantly influence the properties, structure, and functional activity of polysaccharides. Consequently, the selection of extraction methods must consider various factors, such as biological sources, target requirements, and potential interfering substances [31,32]. The extraction and purification technologies of NAPs are shown in Figure 2.

### 2.1. Extraction and Preparation of NAPs

The methods for obtaining NAPs can be broadly categorized into three approaches: extraction from natural or artificially cultivated *N. aurantialba* fruiting bodies; extraction from fermentation-cultured mycelium or fermentation broth; and extraction from fermentation-cultured *N. aurantialba* spore fermentation broth [33,34,35]. Table 1 presents the main extraction techniques of NAPs along with their advantages and disadvantages.

#### 2.1.1. Extraction from Fruiting Bodies

Extraction from natural or artificially cultivated *N. aurantialba* fruiting bodies is the predominant method for obtaining NAPs. Over the years, researchers have optimized and innovated various extraction techniques and processes [29,31,43]. These techniques primarily include hot water extraction (HWE), enzyme-assisted extraction (EAE), ultrasound-assisted extraction (UAE), and alkali-assisted extraction (AAE). These methods are applicable not only to polysaccharide extraction from fruiting bodies but also to mycelial sources [43,44,45].

Hot water extraction (HWE) is the most traditional and commonly used method for polysaccharide extraction. It is based on the principle that most polysaccharides have high solubility in hot water and are more stable. HWE has the advantages of simple operation steps, minimal equipment requirements, and preservation of polysaccharide bioactivity, making it the preferred method of polysaccharide extraction for most edible mushrooms [31,44]. Furthermore, hot water leaching is frequently combined with other extraction methods, such as enzymatic hydrolysis, acid-base hydrolysis, physical treatments, and chemical derivatization, to yield stable and highly biologically active polysaccharides [43]. Yuan et al. extracted TAP-3 from high-quality *N. aurantialba* fruiting bodies by pretreating the corms and then extracting them with hot water at 95 °C for 3 h, repeating the process three times, and achieving a final yield of 50.60% [36]. However, HWE also suffers from low extraction efficiency, long extraction times, and high operating temperatures [32]. For example, Du et al. used 100 °C hot water to extract NAPs from *N. aurantialba* fruiting bodies, resulting in an extraction rate of only 3.84% [37].

The enzyme-assisted extraction (EAE) method utilizes biological enzymes to disrupt the cellulose and cell wall structures of edible fungi, facilitating the dissolution of polysaccharide molecules. This method offers high extraction efficiency, low energy consumption, and mild reaction conditions [46]. Compared to other methods, EAE can effectively increase polysaccharide yield without destroying the polysaccharide structure or bioactivity. However, it is not suitable for industrial production due to enzyme inactivation and high costs [45]. For example, Sun et al. employed cellulase, pectinase, and papain to extract non-starch polysaccharides (NAPs), resulting in the acquisition of TAPS-E fractions. Additionally, Sun et al. used cellulase for NAP extraction, achieving a final extraction rate of 24.95% for NAP-3 [40].

Ultrasound-assisted extraction (UAE) employs the cavitation effect of ultrasound to enhance solvent penetration, disrupt cell walls, and accelerate the release of intracellular components. This method offers several advantages, including short extraction time and extraction rates [46,47]. Several studies have shown that the structure of polysaccharides extracted by UAE can be changed, and the biological activity of polysaccharides extracted without UAE not only has a higher extraction rate, but also has a stronger functional activity [48,49]. For example, Feng et al. found that ultrasound treatment changed the polysaccharide molecular weight and monosaccharide composition ratio of Cucumis sativus polysaccharide (SSU), thus improving its antioxidant and antitumor ability compared to untreated hot water extracted *Cucumis sativus* polysaccharide (SSW) [50]. Huang et al. employed ultrasound extraction to obtain natural active products (NAPs) from the fruiting bodies of the *N. aurantialba*, achieving an extraction rate of 46.36% with high purity [41]. Similarly, Du et al. integrated hot water extraction with ultrasonic extraction methods to isolate TA 2-1 fractions with anti-inflammatory properties [42].

The alkali-assisted extraction (AAE) method is particularly effective for certain acidic polysaccharides or high-molecular-weight polysaccharides, as these polysaccharides have higher solubility in dilute alkaline solutions compared to hot water, significantly increasing the yield of polysaccharides [51,52]. For example, Deng et al. employed this method to isolate the crude polysaccharide CMCP component from the mycelium of the *N. aurantialba* fungus [34]. Zhao et al. extracted acidic polysaccharide components from chicken leg mushrooms using NaOH aqueous solution and further analyzed their structural characteristics and antioxidant activity [53]. Similarly, Yu et al. used EAE, UAE, AAE, and acidic aqueous extract to obtain four polysaccharide fractions (E-NAP, U-NAP, Al-NAP, and Ac-NAP) from the fruiting bodies of *N. aurantialba*, each exhibiting different functional properties [28].

Given the scarcity of natural or artificially cultivated *N. aurantialba* fruiting bodies, research on the extraction of NAPs has been limited [37,41]. The application of more efficient and intelligent emerging technologies, such as microwave-assisted extraction, subcritical water extraction, three-phase extraction, pulsed electric field-assisted extraction, nanoparticle milling extraction, and high-pressure homogenization extraction, may significantly improve polysaccharide yield and enhance the utilization of NAPs.

#### 2.1.2. Mycelium Submerged Fermentation Pathway

Traditional methods for extracting polysaccharides from edible fungi face significant limitations, including long cultivation periods and challenges in maintaining operational consistency. These limitations negatively impact the efficiency of polysaccharide production and the uniformity of their nutritional and bioactive properties, which are crucial for their application as functional food ingredients [34,54]. In contrast, mycelium submerged fermentation is a more efficient method for producing NAPs due to its short cycle time, controllability, traceability, and large-scale production. Moreover, it can significantly enhance the yield, quality, and consistency of polysaccharides by allowing precise control over fermentation parameters, including pH, nutrient replenishment, oxygen levels, fermentation temperature, and fermentation duration [55]. Multiple studies have investigated the optimization of fermentation and extraction conditions for producing NAPs through submerged fermentation of mycelia by modifying the medium formulation and fermentation conditions [56]. Deng et al. cultured the *N. aurantialba* strain on potato dextrose agar (PDA) slant medium for strain acquisition, followed by transferring it to PDA plate medium for rejuvenation. The strain was then transferred to a liquid medium composed of 20% potato, 2% glucose, and 2% agar for fermentation at 28 °C. The mycelial fermentable polysaccharides were successfully obtained using alkaline water extraction [34]. Similarly, Zhang et al. utilized the *T. aurantialba* JSU-05 strain in a 100 mL fermentation setup and successfully obtained a purified polysaccharide, which they named TMP [39].

#### 2.1.3. Spore Fermentation Pathway

Edible fungi from the order Tremellomycetes can produce yeast-like strameno spores, which proliferate by budding in the absence of commensal fungi. This process is easier to regulate compared to traditional mycelial fermentation, making it a viable strategy for large-scale polysaccharide production [57,58]. This method has been successful for the preparation of ginkgo polysaccharides. For example, Zhu et al. produced *Tremella fuciformis* polysaccharides (TFPs) from *T. fuciformis* spores in a 5 L stirred-tank bioreactor under pH-controlled mode, achieving a yield of 4.48 g/L. They further increased the yield of TFPS to 5.80 g/L using a three-stage pH-controlled process [59]. In another study, Sun et al. isolated and purified the *N. aurantialba* spore strain NX-20 and utilized it in fermentation to produce NAPs. The resulting product had the same monosaccharide composition as TAPS obtained by substrate extraction. The NAPS were produced by NX-20 in a 7.5 L fermenter at 25 °C, using tofu wastewater instead of defatted soybean meal as the raw material. The effects of fermentation temperature, duration, initial pH, and inoculum amount on the NAPs yield were systematically evaluated, ultimately achieving a maximum yield of 15.02 ± 0.40 g/L [35]. Subsequent studies have focused on the structural characterization and functional evaluation of the acquired NAPs [58,60]. Spore fermentation is considered a promising technology for NAPs production, offering significant cost savings.

### 2.2. Purification of NAPs

The products obtained through the aforementioned methods are crude polysaccharides mixed with amino acids, proteins, pigments, lipids, inorganic salts, and monosaccharides. These impurities can interfere with the structural analysis and functional activity evaluation of NAPs [61,62,63]. To ensure accurate structural analysis and explore the pharmacological activity of NAPs, the crude polysaccharides must be further separated and purified. Prior to the purification process, the crude polysaccharides must undergo pretreatment. Decolorization methods primarily include activated carbon adsorption, hydrogen peroxide oxidation, and ion exchange. Protein removal techniques encompass the Sevag method, trichloroacetic acid method, and protein digestion method [49,64]. The purification techniques for NAPs mainly include ethanol precipitation, fiber membrane filtration, ion exchange chromatography, and gel column chromatography. Among the various purification methods, graded precipitation of ethanol is the most commonly used technique. In recent years, several novel technologies have been integrated with the ethanol precipitation method to enhance the separation and purification of polysaccharides from *N. aurantialba* [4,37]. Du et al. used an ultrafiltration membrane to complete decolorization and protein removal in one step, then obtained two components, TAPA1 and TAPB1, through ion exchange chromatography and gel permeation chromatography [33,38]. Yuan et al. obtained crude NAPs from *N. aurantialba* fruiting bodies using hot water extraction, followed by dialysis and lyophilization. The TAP-3 fraction was further purified using a DEAE sepharose fast flow column [36]. Sun et al. isolated and purified crude polysaccharides extracted from the conidial fermentation broth using semi-permeable membrane dialysis and the Seveg method, resulting in the acquisition of NAPs [35,58,60]. Peng et al. precipitated polysaccharides using 80% ethanol, followed by further purification through ion exchange chromatography and gel chromatography to isolate the Ta 2-1 fractions [42]. Similarly, Sun et al. extracted polysaccharides from *N. aurantialba* fruiting bodies using ethanol precipitation, followed by dialysis and ion-exchange chromatography to obtain the NAP-3 fractions [40], which were further applied in antihyperglycemic activity studies [65].

## 3. Structural Characterizations of NAPs

Polysaccharides are macromolecular carbohydrates composed of multiple monosaccharides linked by glycosidic bonds. Their structural characterization can be divided into primary and advanced structures. The primary structure refers to the composition of the main chain and branched chains, while the advanced structure involves the conformation of the main chain and non-covalent interactions between polysaccharides [66,67]. As a consequence of the limitations in extraction costs and analytical techniques, the structural characterization of NAPs primarily focuses on the primary structure. This includes the analysis of the total sugar content, molecular weight and distribution, monosaccharide composition and molar ratios, and type of glycosidic bonds [29,30]. The primary techniques involved include nuclear magnetic resonance spectroscopy (NMR), high-performance liquid chromatography (HPLC), gas chromatography-mass spectrometry (GC-MS), and infrared spectroscopy (IR). As shown in Table 2, the structural features of NAPs, such as monosaccharide composition, molecular weight, chemical properties, and other structures, have been summarized [49,68].

### 3.1. Relative Molecular Weight

Molecular weight (M_W_) is a fundamental parameter for characterizing the physicochemical properties of natural plant polysaccharides. A significant correlation has been observed between their pharmacological activities and average molecular weights [71]. Current methods for determining the molecular weight of NAPs include high-performance liquid chromatography (HPLC), high-performance gel chromatography (HPGPC), high-performance gel filtration chromatography (HPGFC), and polyacrylamide gel electrophoresis (PAGE) [18,58]. To date, researchers have successfully extracted and purified NAPs with varying molecular weights. For instance, Fei et al. isolated a low molecular weight heteropolysaccharide (TABP) with a molecular weight of only 5408 Da from the endophytic bacterium *Bacillus* sp. TAB [70]. However, Sun et al. obtained higher molecular weight NAPs-25 and NAPs-30 from the fermentation broth of *N. aurantialba* NX-20 strain, with molecular weights of 2948.0 and 4647.0 kDa, respectively [69]. Interestingly, the same research team used the same strains for fermentation and successfully isolated NAPS-A, NAPS-B, TAPS-E, and TAPS-F with molecular weights of 2924.0, 1763.0, 1130.4, and 2924.6 kDa, respectively [35,58]. Additionally, Du et al. isolated two fractions from the fruiting bodies of *N. aurantialba*, TAPA1 and TAPB1, respectively, with molecular weights of 1350 and 760 kDa [33,38]. These results demonstrate that even when the same source strains are employed for fermentation or extraction of polysaccharides, significant differences in the obtained products can still exist. This variation in M_W_ may be attributed to the diverse sources of NAPs, as well as differences in extraction and purification methods.

### 3.2. Monosaccharide Composition

Natural plant polysaccharides are typically composed of monosaccharides with varying chemical structures linked by glycosidic bonds, resulting in a wide diversity of polysaccharide structures [72]. Consequently, a detailed analysis of monosaccharide composition is crucial to reveal the structural diversity and pharmacological activities of these polysaccharides [73]. Current methodologies for monosaccharide composition determination include high-performance liquid chromatography (HPLC), Fourier transform infrared spectroscopy (FT-IR), capillary electrophoresis (CE), and gas chromatography-mass spectrometry (GC-MS) [68,74]. Comprehensive studies have shown that NAPs are composed of various monosaccharides, including Galactose (Gal), Mannose (Man), Epichitosamine (Man-N), Glucose (Glc), Fructose (Fru), Glucosamine hydrochloride (GlcN), Glucuronic acid (GlcA), Rhamnose (Rha), Galacturonic acid (GalA), Arabinose (Ara), and Xylose (Xyl) [75]. For example, Sun et al. used the spore fermentation method to produce NAPs as an alternative to traditional fruiting body extraction. They isolated and purified four polysaccharide components from the NX-20 strain of *N. aurantialba*: NAPS-A, NAPS-B, TAPS-E, and TAPS-F. Among them, NAPS-A and NAPS-B are composed of mannose (Man), xylose (Xyl), glucuronic acid (GlcA), glucose (Glc), and galactose (Gal), while TAPS-E and TAPS-F consist of five monosaccharides: Man-N, Man, GlcA, Glc, and Xyl, with varying proportions [58]. Earlier, Du et al. identified only three monosaccharides, namely Man, Xyl, and GlcA, from the NAPs of the *N. aurantialba* fruiting bodies, specifically TAPA1 (Man:Xyl:GlcA = 5:4:1) and TAPB1 (Man:Xyl:GlcA = 3.1:2.9:1.2) [38]. In a subsequent study, Huang et al. employed ultrasonication to extract polysaccharides from the fruiting bodies, which were designated as NAPs. The monosaccharide composition of these NAPs included Man (59.04%), Xyl (23.89%), GlcA (14.07%), GalA (2.12%), and Glc (0.76%). Additionally, some other NAPs exhibited unique monosaccharide compositions, such as Fru and GlcN found in TA 2-1 and TABP, respectively [41].

### 3.3. Structural Characteristics

The chemical structures of polysaccharides, such as the length of the sugar chain, linkage types, degree of branching, and types of monosaccharide residues, collectively determine their biological activities [29,46,47,48]. Consequently, an in-depth investigation of the chemical structure of NAPs is essential for elucidating their physiological functions. Current methods for studying the chemical structure of NAPs include high-performance gel permeation chromatography (HPGPC), high-performance liquid chromatography (HPLC), gas chromatography-mass spectrometry (GC-MS), Fourier-transform infrared spectroscopy (FT-IR), nuclear magnetic resonance (NMR), atomic force microscopy (AFM), transmission electron microscopy (TEM), and scanning electron microscopy (SEM), and so on [43,49]. The methods not only determine the type and configuration of glycosidic bonds in NAPs, but also facilitate the accurate analysis of substituent types, repeating units, and the linkage order within polysaccharide chains [63]. NMR is a common technology employed for the structural analysis of polysaccharides, as it can identify the types of glycosidic bonds, the specific monosaccharides present, and the sequence of glycosidic linkages through chemical shifts. In contrast, FT-IR is typically used to ascertain the types of polysaccharides and some aspects of their configurations via characteristic absorption peaks. Complementing these techniques, LC-MS/MS and MALDI-TOF/MS provide direct sequence evidence by analyzing oligosaccharide fragmentation patterns, enabling the determination of glycosidic linkage sequences and branching points with high sensitivity, which may play a crucial role in the future structural analysis of NAPs [48,51]. Additionally, SEM and AFM are commonly engaged for analyzing the surface topography of polysaccharides [72,74]. Significant progress has been made in the structural characterization of NAPs. Du et al. structurally characterized two NAPs separately, identifying four residues in TAPA1 as →3)-α-D-Manp-(1→3)-α-D- Manp-(1→3)-α-D-Manp-(1→, 4-β-D-Manp-(1→3)-β-D-Xylp-(1→4)-β-D-ClcAp-(1→2)-α-D-Manp-(1→, α-D-Manp-(1→4)-β-D-Xylp-(1→2)-α-D-Manp-(1→,β-D-Xylp-(1→2)-β-D-Xylp-(1→4) -α-D-Manp-(1→; Meanwhile three residues were detected in TAPB1 as →3)-α-D -Manp-(1→3)-α-D-Manp-(1→3)-α-D-Manp-(1→,β-D-ClcAp-(1→3)-β-D-Xylp-(1→2)-α-D-Manp-(1→, β-D-Xylp-(1→2)-β-D-Xylp-(1→4)-α-D-Manp-(1→ [38]. With advancements in technology and a deeper understanding of polysaccharide structures, researchers have successfully identified not only the types of sugar residues but also their respective proportions. For instance, in the study by Sun et al., the proportions of various residues in NAPS-25 were reported as follows: 2,3,4-Me3-Xylp:2,4-Me2-Xylp:3,4-Me2-Xylp:2,3,4,6- Me4-Manp:2,3,6-Me3-GlcAp:2,4,6-Me3-Manp:6-Me-Manp = 10.27:7.07:13.58:31.00:2.45:20.05:15.58. In contrast, the ratios of several residues in NAPS-30 were 2,3,4-Me3-Xylp:2,4-Me2-Xylp:3,4-Me2-Xylp:2,3,4,6-Me4-Manp:2,3,6-Me3-GlcAp:2,4,6-Me3-Manp:6-Me-Manp = 8.69:8.17:13.95:26.76:2.85:20.62:18.96 [69]. In a subsequent study, the structure of NAPS-A was elucidated through methylation analysis and NMR, revealing sugar residues such as →3)-α-D-Manp, →2,3,4)-α-D-Manp-(1→, →3)-α-D-Manp-(1→, β-D-Manp-(1→, →2)-β-D-Xylp-(1→, β-D-Xylp-(1→, and →3)-β-D-Xylp-(1→. SEM and TEM were used to analyze the microstructure of NAPS-A, while XRD analysis showed that the polysaccharide exhibited bun-shaped non-crystalline structures [58].

The activity-structure relationship of NAPs remains underexplored. Sun et al. found that NAPS-A (4647 kDa) preferentially binds with water molecules in solution, reducing free water and ice crystal formation, thereby enhancing antifreeze quality and making it a valuable additive for frozen foods [58]. Conversely, lower molecular weight TABP (5408 Da) effectively promotes microbial growth as a prebiotic [70]. Du et al. proposed that sulphonation and acetylation of TAPA1 enhance its immunostimulatory and antioxidant activities, respectively [38]. Additionally, Yuan et al. demonstrated that the immunomodulatory activity of TAP-3 correlates with its chain length, with the low molecular weight dTAP-3a fragment showing superior activity [36].

## 4. Biological Activity of NAPs

**Figure 3:**
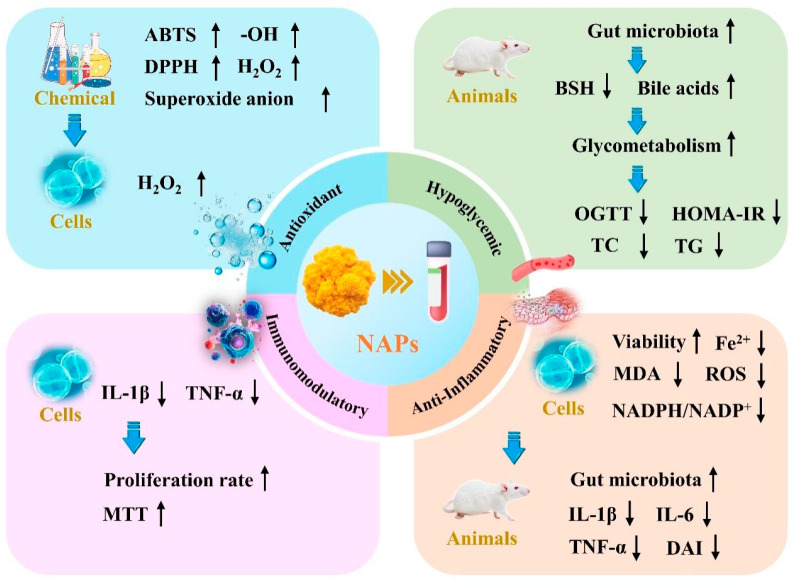
Overview of the key biological activities and mechanisms of action of NAPs exhibiting antioxidant, hypoglycemic, anti-inflammatory, and immunomodulatory activities. The upward arrow represents an increase in activity or content, while the downward arrow represents a decrease in activity or content.

As a well-known fungus recognized for its dual role as both a food source and medicinal agent, its traditional pharmacological effects are extensively documented in classical Chinese medicine texts. For instance, the “Record of Famous Doctors” (5th–6th Century AD, Tao Hongjing, Late Liang to Early Tang Dynasty) notes that *N. aurantialba* benefits brain health and helps dispel cold effects. In addition, the “Compendium of Materia Medica” (Li Shizhen, 1578 AD, Ming Dynasty) from the Ming dynasty records its use in treating various diseases, highlighting its efficacy in moistening the lungs, relieving cough, preserving liver function, and tonifying the kidneys [1,5]. With the increasing attention to the physiological efficacy of traditional medicinal and food products, the modern chemical composition of *N. aurantialba* has been thoroughly analyzed, confirming its richness in dietary fiber, polyphenols, active peptides, polysaccharides, and other functional substances. Among these, polysaccharides are identified as the most significant active components in *N. aurantialba*, demonstrating various beneficial effects, including antioxidant, hypoglycemic, immune regulation, intestinal microbiota modulation, anti-tumor, and anti-inflammatory properties [23,36,37]. Figure 3 illustrates the main functional activities and related mechanisms of NAPs, while Table 3 summarizes the key research progress on their physiological activities.

### 4.1. Antioxidant Activity

Oxidative stress is a disorder associated with an imbalance in redox mechanisms, typically resulting from an excess of reactive oxygen species (ROS) or reactive nitrogen species (RNS) in the body, which can cause cellular damage through direct or indirect means [80,81]. NAPs exhibit potent antioxidant activity and can protect cells and tissues from damage by effectively scavenging free radicals and alleviating oxidative stress. Both in vivo and in vitro studies have investigated the antioxidant activity of NAPs [37,69,76]. In the study conducted by Du et al., TAPA1 and TAPB1, derived from fruiting bodies, demonstrated extremely strong superoxide anion and H_2_O_2_ scavenging capabilities in vitro. In a subsequent study, a PC12 cell model of oxidative injury induced by H_2_O_2_ was treated with a 500 µg/mL solution of TAPA1, resulting in a cell survival rate increase from 56.10% to 157.63% [23,33,76]. Similarly, Huang et al. reported that NAPs obtained from substrates effectively scavenged ABTS, DPPH, and superoxide anion radical [41]. In an in vivo study, NAP-3 reduced malondialdehyde (MDA) levels in the serum of hyperlipidemic mice by regulating gene expression while increasing catalase (CAT) and glutathione (GSH) levels, thereby alleviating oxidative stress in these mice [40]. It is noteworthy that NAPs obtained from tansy spore fermentation have similar effects. Sun et al. also demonstrated that NAPS-A and NAPS-B exhibited strong scavenging activities against ABTS, DPPH, OH, and superoxide anion radicals, while NAPS-25 and NAPS-30 effectively scavenged DPPH and superoxide anion radicals [58,69]. Furthermore, Zhang et al. reported that TMP extracted from mycelium significantly increased total antioxidant capacity (T-AOC), catalase (CAT), superoxide dismutase (SOD), and glutathione (GC) levels, while reducing malondialdehyde (MDA) content in mouse tissues, effectively alleviating oxidative stress in type 2 diabetic rats [39].

### 4.2. Hypoglycemic Activity

Type 2 diabetes mellitus (T2D) is characterized by a progressive and uneven loss of insulin secretion from pancreatic β-cells, often following insulin resistance (IR). This condition is a component of the metabolic syndrome and accounts for approximately 96% of diabetes mellitus cases [82,83,84]. Polysaccharides derived from various edible fungi have been shown to effectively improve hyperglycemia and diabetes through glucose metabolism regulation and enhanced insulin sensitivity and resistance [85,86]. For example, Sun et al. demonstrated that NAP-3 improved HepG2 cell damage by increasing glucose consumption and reducing ROS generation. In a T2D mouse model, the combination of NAP-3 and metformin showed significant hypoglycemic activity, effectively reducing body weight, serum insulin level, glucose tolerance, and insulin tolerance [40]. Their follow-up study further revealed that NAP-3 enhanced metformin’s efficacy on lipid and glucose metabolism in T2D mice through a gut microbiome-dependent mechanism. This enhancement amplified the effect of metformin on glucose metabolism in T2D mice via the gut microbial-bile acid-nuclear receptor-GLP-1 interaction axis, effectively ameliorating diabetes [65]. In another study, TAP effectively reduced plasma glucose levels by increasing insulin levels in mice, with further studies demonstrating TAP improved diabetes by increasing hepatic glucose metabolism through elevated activities of glucokinase, hexokinase and glucose 6-phosphate dehydrogenase in vivo [77,78,79]. Therefore, NAPs represent a promising therapeutic option for diabetes treatment.

### 4.3. Immunomodulatory Activity

Immunomodulation is crucial for maintaining the balance of the immune system, ensuring effective defense against pathogens while preventing host tissue damage [87]. NAPs, as natural edible fungus polysaccharides, have significant immunomodulatory functions [5]. Yuan et al. reported that TAP-3 significantly enhanced macrophage secretion of nitric oxide (NO), interleukin-1 beta (IL-1β), and tumor necrosis factor-alpha (TNF-α), functioning as an immunostimulatory agent [36]. Du et al. compared the immunostimulatory activities of crude, semi-purified, and purified polysaccharides extracted from *N. aurantialba* using mouse spleen lymphocytes. The results confirmed that the purified polysaccharide TAPA1 exhibited the strongest immunomodulatory activity, with concentrations of 50, 200, and 500 μg/mL increasing mouse spleen lymphocytes proliferation rates to 339.67%, 484.10%, and 593.98%, respectively [37,38].

### 4.4. Other Activity

In addition to the above activities, NAPs possess other pharmacological activities, such as anti-inflammatory, anti-tumor, regulation of gut microbiota, hypolipidemic activity, and life-extension properties. Anti-tumor activity is usually associated with immune modulation, and NAPs have demonstrated some anti-tumor effects [36,38,87]. Lee et al. reported that crude polysaccharides extracted from the fruiting bodies of *N. aurantialba* exhibited no significant toxicity to RAW 309 CR.1 and Sarcoma 180 cells but extended the lifespan of Sarcoma 180-inoculated homozygous mice by 11.1% to 66.7% through immune response modulation [88]. Oxidative stress is closely linked to inflammatory responses, and NAPs with strong antioxidant activity play a crucial role in preventing certain inflammatory conditions [80,81]. Peng et al. demonstrated that the small molecule TA 2-1 significantly reduced ulcerative colitis symptoms in a mouse model by regulating intestinal flora and inhibiting iron apoptosis in epithelial cells [42]. Disturbances in gut microbiota can induce various metabolic diseases, such as hyperlipidemia, diabetes, inflammatory responses, and cancer [89,90]. Sun et al. and Peng et al. proved that NAP-3 and TA 2-1 alleviated T2D and ulcerative colitis in mice by modulating intestinal flora [42,65]. Fei et al. reported that TABP significantly promoted the growth of *Lacticaseibacillus paracasei* and *Lacticaseibacillus rhamnosus* strains (growth rates exceeding 90%) [70]. Zhang et al. illustrated that TMP effectively regulated lipid metabolism, reducing cholesterol, phospholipids, and triglycerides in the plasma of diabetic rats [39]. Overall, NAPs exhibit various biological activities, positioning them as a promising category of active ingredients derived from edible mushroom sources with broad applications.

## 5. Application of NAPs

In recent years, the increasing emphasis on health has driven a growing interest in natural products. Nutrients derived from plants or edible fungi, which are known for their beneficial effects, hold significant potential in fields such as functional foods, clinical medicine, and cosmetics [25,27,91,92]. *N. aurantialba*, with its superior functional characteristics, has gained recognition for its significant value in production and application, leading to wide-spread utilization across various fields [4,23]. China has a long history of consuming *N. aurantialba*, which is regarded as a functional raw material for both culinary and medicinal purposes. Compared to other regions, its applications in China are more diverse, and it has been widely accepted as a food ingredient [1,5,93]. For example, Cheng et al. investigated the impact of *N. aurantialba* powder on the gelatinization characteristics of starch and subsequently prepared starch jelly [94]. Feng et al. reported the preparation of an *N. aurantialba* bread, which significantly improved the sensory acceptability of the bread and effectively inhibited glucose release. This finding plays an important role in the development and application of starch-based products [13]. Zhang et al. fermented cigar filler leaves using *Tremella aurantialba* SCT-F3 (CGMCC No. 23831), thereby enhancing the sensory quality of cigar filler leaves [95]. Similarly, NAPs have been utilized in the development of a variety of functional foods and cosmetics. Sun et al. reported that the macromolecular polysaccharide NAPS-A exhibited significant antioxidant and antifreeze properties, providing a chemical and biological basis for the development of novel functional food additives and antifreeze agents [58]. Owing to its excellent water retention properties, *N. aurantialba* is commonly used in the development of various cosmetic products. As the functional value of NAPs is gradually realized and new preparation processes emerge that significantly reduce their production cost of NAPs. NAPs are expected to be further utilized in the development of more products, with broad application prospects and high economic value.

## 6. Conclusions and Future Perspectives

Natural polysaccharides, including NAPs, are emerging as promising candidates for functional food supplements and pharmaceutical development due to their abundant sources, high safety profile, minimal side effects, and diverse bioactivities. This review comprehensively examines the extraction, purification, structural characterization, bioactivity and application of NAPs in food, cosmetics and other industries, highlighting their practical value and research potential. NAPs exhibit a wide range of functional activities, including antioxidant, hypoglycemic, immunomodulatory, intestinal flora regulation, antitumor, and anti-inflammatory effects. These bioactivities are influenced by various factors, including raw material sources, extraction methods, purification techniques, molecular weight, structural characteristics, and chemical modifications, which present both opportunities and challenges for their application in disease management and industrial production.

Despite progress in NAPs research and the registration of *N. aurantialba* as a new cosmetic ingredient, several challenges remain. These include challenges in its extraction and purification, structural analysis, mechanism of action, clinical validation and industrial application, which need to be further explored in depth. Most studies on NAPs are confined to laboratory settings, with a significant gap remaining between experimental findings and practical industrial applications. Optimizing industrial production technologies is thus a priority. The efficiency of polysaccharide extraction and purification is a critical bottleneck. The biological activity of NAPs is closely related to their structural characteristics, and different extraction methods yield polysaccharides with varying structures. Therefore, optimizing extraction and purification procedures based on specific requirements is essential. Structural analysis of NAPs remains a significant hurdle. While current research has focused on monosaccharide composition and major polysaccharide residues, in-depth analysis of the backbone structure and specific structural features is lacking. This gap constrains the investigation of structure-function relationships in NAPs. The mechanisms underlying NAPs’ biological effects, such as hypoglycemic and hypolipidemic actions, are not fully understood. Moreover, while in vitro and animal studies are prevalent, clinical research remains limited.

Given the substantial industrial and clinical potential of NAPs, future research should focus on several key areas: (1) innovative preparation processes, including developing novel production methods such as strain screening and cultivation to reduce industrial costs; (2) integrated mechanistic studies, combining in vitro, animal, and clinical experiments to elucidate the mechanisms of NAPs’ biological activities; (3) nano delivery systems, exploring new technologies to enhance NAPs’ bioavailability; and (4) artificial intelligence integration, using AI to optimize interactions between NAPs and other food components, thereby reducing application costs and maximizing functional value. In summary, addressing these challenges and harnessing emerging technologies will be essential for advancing the research and applications of NAPs in functional foods, pharmaceuticals, and other industries.

## Figures and Tables

**Table 1:** The comparison of different extraction techniques of NAPs.

Techniques	Principle	Extraction Conditions						Evaluation	References
		Sources	Time	Repetition	Temperature	Solid–Liquid Ratio	Yield		
HWE	NAPs have a higher solubility in hot water and a stable structure	Fruiting bodies	3 h	Thrice	95 °C	1:40	50.60%	Advantages: Simple operation and equipment, low cost; Disadvantages: Long extraction time, low extraction rate, high temperature.	[36]
		Fruiting bodies	100 min	Thrice	100 °C	1:10	3.84%		[37]
		Fruiting bodies	2 h	Thrice	100 °C	1:10	-		[38]
		Mycelium	4 h	Thrice	100 °C	1:10	-		[39]
EAE	Biological enzymes can destroy the cell wall structure and accelerate the dissolution of NAPs	Fruiting bodies	45 min	Once	55 °C	1:15	-	Advantages: Mild conditions, low energy consumption, high efficiency; Disadvantages: expensive and prone to inactivation.	[35]
		Fruiting bodies	2 h	Thrice	45 °C	1:40	24.95%		[40]
UAE	Break down cell walls, and accelerate the precipitation rate of intracellular components	Fruiting bodies	32 min	Once	-	1:49	46.36%	Advantages: High efficiency, low energy consumption, simple operation.	[41]
		Fruiting bodies	4 h	Once	75 °C	1:20	-		[42]
AAE	Acidic polysaccharides have a higher solubility in alkaline solutions	Mycelium	4–6 h	Once	25 °C	-	1.53%	Advantages: High efficiency, high extraction rate; Disadvantage: Only suitable for acidic polysaccharides.	[34]
EAE/UAE	Increase extraction efficiency	Fruiting bodies	1 h	Once	80 °C	1:80	50.15%	Advantages: High extraction rate, low energy consumption; Disadvantages: High cost, complex operation steps	[28]

Note: HWE, hot water extraction; EAE, enzyme-assisted extraction; UAE, ultrasound-assisted extraction; AAE, alkali-assisted extraction; EAE/UAE, enzyme and ultrasonic-assisted extraction; -, not determined.

**Table 2:** Molecular weight, monosaccharide composition, and structure determination of NAPs.

Name	Carbohydrate Content	Molecular Weight (kDa)	Monosaccharide Composition	Structural Features	Analysis Technique	References
TAPS-E	-	1130.4	Man-N:Man:GlcA:Glc:Xyl = 3.3:52.0:4.1:2.4:1.8:36.4	-	HPLC, GPC, FT-IR	[35]
TAPS-F	-	2924.6	Man-N:Man:GlcA:Glc:Xyl = 8.7:47.4:2.0:3.4:1.0:37.5	-		
NAPS-A	93.4%	2924.0	Man:Xyl:GlcA:Glc:Gal = 59.75:31.73:4.20:2.51:1.81	The structure is inferred as presented below: →3)-*α*-DManp, →2,3,4)-*α*-D-Manp-(1→, →3)-*α*-D-Manp-(1→, *β*-DManp-(1→, →2)-*β*-D-Xylp-(1→, *β*-D-Xylp-(1→, and →3)-*β*-DXylp-(1→ sugar residues	GPC, HPLC, FT-IR, GC-MS, NMR, SEM, AFM, XRD, DSC, TG	[58]
NAPS-B	90.6%	1763.0	Man:Xyl:GlcA:Glc:Gal = 38.17:26.40:7.08:7.79:20.56	-		
NAPS-25	91.0%	2948.0	Man:Xyl:GlcA:Glc:Gal = 59.17:32.26:4.42:1.39:2.76	2,3,4-Me3-Xylp:2,4-Me2-Xylp:3,4-Me2-Xylp:2,3,4,6-Me4-Manp:2,3,6-Me3-GlcAp:2,4,6-Me3-Manp:6-Me-Manp = 10.27:7.07:13.58:31.00:2.45:20.05:15.58	HPLC, FT-IR, SEM	[69]
NAPS-30	90.9%	4647.0	Man:Xyl:GlcA:Glc:Gal = 61.99:30.06:4.65:1.44:1.86	2,3,4-Me3-Xylp:2,4-Me2-Xylp:3,4-Me2-Xylp:2,3,4,6- Me4-Manp:2,3,6-Me3-GlcAp:2,4,6-Me3-Manp:6-Me-Manp = 8.69:8.17:13.95:26.76:2.85:20.62:18.96		
TAPA1	98.7%	1350.0	Man:Xyl:GlcA = 5:4:1	The sequences of 4 residues are as follows: →3)-*α*-D-Manp-(1→3)-*α*-D-Manp-(1→3)-*α*-D-Manp-(1→,4-*β*-D-Manp-(1→3)-*β*-D-Xylp-(1→4)-*β*-D-ClcAp-(1→2)-*α*-D-Manp-(1→,*α*-D-Manp-(1→4)-*β*-D-Xylp-(1→2)-*α*-D-Manp-(1→,*β*-D-Xylp-(1→2)-*β*-D-Xylp-(1→4)-*α*-D-Manp-(1→	GPC, HPAEC-PAD, FT-IR, NMR	[38]
TAPB1	97.6%	760.0	Man:Xyl:GlcA = 3.1:2.9:1.2	The sequences of 3 residues are as follows: →3)-*α*-D-Manp-(1→3)-*α*-D-Manp-(1→3)-*α*-D-Manp-(1→, *β*-D-ClcAp-(1→3)-*β*-D-Xylp-(1→2)-*α*-D-Manp-(1→, *β*-D-Xylp-(1→2)-*β*-D-Xylp-(1→4)-*α*-D-Manp-(1→	FT-IR, HPAEC-PAD, GPC, GC-MS, NMR	[33]
NAPS-A	93.4%	2930.0	Man:Xyl:GlcA:Glc:Gal = 1.08:0.57:0.08:0.01:0.01	-	GPC, HPLC, FT-IR, SEM	[60]
TAP-3	61.8%	624.0	Man:Xyl:GlcA = 27.31:9.02:8.86	7 linkage forms: (1→3)-linked Xylp, terminal Xylp, (1→4)—linked GlcpA, terminal Manp, (1→3)—linked Manp, (1→ 2,3)—linked Manp and (1→ 2)—linked Manp.	HPGPC, GC-MS, NMR, SEM	[36]
NAP-3	93.4%	428.0	Man:Rha:Xyl = 67.39:7.87:22.91	5 types of residues: *β*-1,2,3-D-Manp, terminal *β*-DXylp, *β*-1, 4-D-Glcp, *β*-1,3-D-Manp, *β*-1, 4-D-Rhap	HPGPC, FT-IR, XRD, HPLC, SEM, NMR	[40]
NAP	89.9%	915.0	Man:Xyl:GlcA:GalA:Glc = 59.04:23.89:14.07:2.12:0.76	-	HPLC-SEC, FT-IR	[41]
TA 2-1	-	127.0	Man:Xyl:GlcA:Glc:Fru:Rha = 59.2:23.1:13.9:1.6:1.7:0.4	1, 3-Man with branch chains of T-Xylp, 1,3Xylp, 1,4-GlcAp, and T-Manp at its O-2 position	HPLC-SEC, FT-IR, GC-MS	[42]
TABP	-	5.4	Ara:GlcN:Gal:Glc:Man = 0.073:0.145:0.406:0.182:0.195	Linkage types: 1,5-Araf, 1,4- linked-GlcpN, 1,4-linked-Galp, 1,4-linked-Manp, 1,6-linked-Manp, 1,4,6-linked-Galp, 1,2,6-linked-Manp, and 1,3,5-Araf	HPGPC, HPAEC, FT-IR, GC-MS, NMR	[70]

Notes: HPLC, high-performance liquid chromatography; HPLC-SEC, high-performance liquid chromatography-size exclusion chromatography; GC-MS, gas chromatography-mass spectrometer; FT-IR, Fourier transform infrared spectroscopy; GPC, gel permeation chromatography; CD, circular dichroism; XRD, X-ray diffraction spectrum; NMR, nuclear magnetic resonance spectroscopy, DSC, differential scanning calorimetry; TG, thermogravimetry; HPAEC, high-performance anion-exchange chromatography; HPAEC-PAD, high-performance anion-exchange pulsed-amperometric detection chromatography; HPGPC, high-performance gel permeation chromatography; Gal, galactose; Man, mannose; Man-N, epichitosamine; Glc, glucose; Fru, fructose; GlcA, glucose acid; Rha, rhamnose; GlcN, glucosamine hydrochloride; GalA, galacturonic acid; Ara, arabinose; Xyl, xylose; “-”, not determined.

**Table 3:** Research on the physiological activities of NAPs.

Bioactivities	Sources	Models	Measurement Indicators	References
Antioxidant activity	Basidiospore	Chemical determination	ABTS, DPPH, OH, and superoxide anion radical scavenging.	[58]
	Basidiospore	Chemical determination	DPPH and superoxide anion radical scavenging.	[69]
	Fruiting bodies	Chemical determination	Superoxide anion and H_2_O_2_ scavenging.	[76]
		Oxidative injury PC12 cells	Cell viability.	
	Fruiting bodies	Chemical determination	Superoxide anion and H_2_O_2_ scavenging.	[33]
	Fruiting bodies	Chemical determination	ABTS, DPPH, and hydroxyl radical scavenging.	[41]
Hypoglycemic activity	Fruiting bodies	Type 2 diabetic mice	Weight, fasting blood glucose, food consumption; Metabolic phenotyping: OGTT, HOMA-IR; LP-1, BSH; Enzymes activity: CAT, GSH-Px, SOD, MDA;	[40]
		Human HepG2 cells	Glucose consumption, cytotoxicity assay, ROS	
	Fruiting bodies	Type 2 diabetic mice	Weight, fasting blood glucose, food consumption; Metabolic phenotyping: OGTT, ITT, HOMA-IR, TC, TG; Liver function: AST, ALT; Antioxidant enzymes activity: CAT, GSH-Px, SOD, MDA; Histopathology: H&E staining; Intestinal permeability: D-LA, DAO; Gut microbiota: 16s rRNA gene sequencing; Genetic expression: RT-PCR.	[65]
	Fruiting bodies	STZ-induced diabetic mice	Key indicators in glycometabolism pathway: phofructokinase, glycogen, and plasma cholesterol.	[77]
	Fruiting bodies	ddY mice	Plasma glucose, water and food consumption.	[77,78]
	Fruiting bodies	Type 2 diabetic mice	Weight, food and water consumption; Plasma insulin, plasma lipid, and lipid in feces.	[79]
Immunomodulatory activity	Fruiting bodies	RAW264.7 cells	Cell viability: MTT; Inflammation: IL-1*β* and TNF-*α*	[36]
	Fruiting bodies	C57BL/6 male mice spleen cells	Proliferation rate	[37]
	Fruiting bodies	C57BL/6 male mice spleen cells	Proliferation rate	[38]
Anti-colitis activity	Fruiting bodies	Erastin-induced Caco-2 cells	Viability, Fe^2+^, MDA, ROS, NADPH/NADP^+^	[42]
		DSS-induced colitis mice	Apparent indicators: body weight loss, fecal consistency, fecal blood test scores, and DAI; Pathological evaluation: H&E staining, immunohistochemistry, and immunofluorescence staining; Inflammatory cytokine levels: IL-6, TNF-*α*, and MCP-1.	
Prebiotic activity	Fruiting bodies	*Lacticaseibacillus paracasei*, *Lacticaseibacillus rhamnosus*	Prebiotic activity: growth curve.	[70]
Hypolipidemic activity	Mycelium	Type 2 diabetic rats	blood glucose, weight, food consumption, plasma cholesterol, plasma phospholipids, plasma triglyceride, T-AOC, CAT, SOD, MDA, GSH-Px, GC	[39]

Note: CAT, Catalase; GSH-Px, glutathione peroxidase; SOD, superoxide dismutase; MDA, malondialdehydes; NADPH, nicotinamide adenine dinucleotide phosphate hydrogen; NADP+, nicotinamide adenine dinucleotide phosphate; OGTT, oral glucose tolerance test; HOMA-IR, homeostasis model assessment of insulin resistance index; ITT, insulin tolerance test; TC, total cholesterol; TG, total triglyceride; AST, aspartate transaminase; ALT, alanine transaminase; D-LA, D-lactic acid; DAO, diamine oxidase; H&E, hematoxylin-eosin; BSH, bile salt hydrolase; ABTS, 2,2′-azino-bis(3-ethylbenzothiazoline-6-sulfonic acid); DPPH, 2,2-diphenyl-1-picrylhydrazyl; STZ, streptozotocin; DAI, disease activity index; T-AOC, total antioxidant activities; GC, glutathione reductase, MTT, 3-(4,5-Dimethylthiazol-2-yl)-2,5-diphenyltetrazolium bromide; IL-1β, interleukin-1β; IL-6, interleukin-6; TNF-α, tumor necrosis factor-α; MCP-1, monocyte chemoattractant protein-1; WB, Western blot; RT-PCR, reverse transcription-polymerase chain reaction.

## Data Availability

Not applicable.

## Abbreviations

The following abbreviations are used in this manuscript:

AAE	Alkali-assisted extraction
ABTS	2,2′-azino-bis (3-ethylbenzothiazoline-6-sulfonic acid)
ALT	Alanine transaminase
Ara	Arabinose
AST	Aspartate transaminase
BSH	Bile salt hydrolase
CAT	Catalase
CD	Circular dichroism
CE	Capillary electrophoresis
DAI	Disease activity index
DAO	Diamine oxidase
D-LA	D-lactic acid
DPPH	2,2-diphenyl-1-picrylhydrazyl
DSC	Differential scanning calorimetry
EAE	Enzyme-assisted extraction
EAE/UAE	Enzyme and ultrasonic-assisted extraction
Fru	Fructose
FT-IR	Fourier transform infrared spectroscopy
Gal	Galactose
GalA	Galacturonic acid
GC	Glutathione reductase
GC-MS	Gas chromatography-mass spectrometer
Glc	Glucose
GlcA	Glucose acid
GlcN	Glucosamine hydrochloride
GPC	Gel permeation chromatography
GSH	Glutathione
GSH-Px	Glutathione peroxidase
H&E	Hematoxylin-eosin
HOMA-IR	Homeostasis model assessment of the insulin resistance index
HPAEC	High-performance anion-exchange chromatography
HPAEC-PAD	High-performance anion-exchange pulsed-amperometric detection chromatography
HPGFC	High-performance gel filtration chromatography
HPGPC	High-performance gel permeation chromatography
HPLC	High-performance liquid chromatography
HPLC-SEC	High-performance liquid chromatography-size exclusion chromatography
HWE	Hot water extraction
IL-1β	Interleukin-1β
IL-6	Interleukin-6
ITT	Insulin tolerance test
Man	Mannose
Man-N	Epichitosamine
MCP-1	Monocyte chemoattractant protein-1
MDA	Malondialdehydes
MTT	3-(4,5-Dimethylthiazol-2-yl)-2,5-diphenyltetrazolium bromide
M_W_	Molecular weight
NADP+	Nicotinamide adenine dinucleotide phosphate
NADPH	Nicotinamide adenine dinucleotide phosphate hydrogen
*N. aurantialba*	*Naematelia aurantialba*
NAPs	*Naematelia aurantialba* polysaccharides
NMR	Nuclear magnetic resonance spectroscopy
NO	Nitric oxide
OGTT	Oral glucose tolerance test
PAGE	Polyacrylamide gel electrophoresis
PDA	Potato dextrose agar
Rha	Rhamnose
RNS	Reactive nitrogen species
ROS	Reactive oxygen species
RT-PCR	Reverse transcription-polymerase chain reaction
SOD	Superoxide dismutase
SSU	Cucumis sativus polysaccharide extracted by the ultrasound-assisted method
SSW	Cucumis sativus polysaccharide extracted by the hot water method
STZ	Streptozotocin
*T. aurantialba*	*Tremella aurantialba*
T2D	Type 2 diabetes mellitus
T-AOC	Total antioxidant activities
TC	Total cholesterol
TFPs	Tremella fuciformis polysaccharides
TG	Total triglyceride
TNF-α	Tumor necrosis factor-α
UAE	Ultrasound-assisted extraction
UC	Ulcerative colitis
WB	Western blot
XRD	X-ray diffraction spectrum
Xyl	Xylose
